# Rewriting the narrative: resilience of youth in the HIV response

**DOI:** 10.1002/jia2.70019

**Published:** 2025-07-18

**Authors:** Fletcher Chiu, Kairon Liu, Ismail Senyonga

**Affiliations:** ^1^ Persons with HIV/AIDS Rights Advocacy Association of Taiwan Taipei Taiwan; ^2^ Taiwan HIV Story Association; Independent Artist Taipei Taiwan; ^3^ Independent Artist Kampala Uganda

1

The United Nations defines “youth” as individuals aged 15–24 years, although some countries extend this range up to 35 years. According to 2024 UNAIDS epidemiologic estimates, young people aged 15–24 years bear a disproportionate burden of new HIV acquisitions, accounting for approximately 28% of all new acquisitions despite representing only 8% of all people living with HIV [1]. As we commemorate International Youth Day 2025, the global HIV response stands at a critical juncture. Yet, amid adversity, youth‐led organizations and young people persist in their fight—not only for survival, but also for dignity, health and a future free from stigma.

Recent policy shifts and funding cuts—especially to the U.S. PEPFAR programme—have severely disrupted youth‐led organizations in low‐ and middle‐income countries, jeopardizing critical HIV prevention, treatment, care and support services for young people. A survey conducted by Y+ Global and partners in early 2025 revealed that 60% of youth‐led organizations had experienced interruptions in delivering core HIV services as a result of these cuts. Despite the setbacks, they have demonstrated remarkable resilience by uniting to urge stakeholders to take action to preserve youth‐led HIV responses. They have also swiftly adapted by exploring alternative funding mechanisms, such as crowdfunding campaigns and partnerships with the private sector [2].

In addition to these challenges to healthcare access and advancing youth leadership, people living with HIV continue to face pervasive stigma, resulting in barriers in the workplace, intimate relationships, community settings and beyond. The 2023 People Living with HIV Stigma Index Global Report found that 85% of individuals living with HIV experience internalized stigma, underscoring the profound psychological impact of the epidemic. Notably, this rate is even higher among young people, with 88% reporting internalized stigma [3]. The growing backlash against Diversity, Equity and Inclusion principles and programmes further exacerbates the potential for stigma and discrimination against HIV and key populations.

In this context, the dual meaning of “ART” as standing for both antiretroviral therapy and artistic creation takes on powerful significance. While ART sustains biological life and can help prevent HIV transmission, art fosters hope and social connection—elements equally essential for thriving with HIV. The HIV Science as Art programme, launched in 2023, has highlighted how art enriches the value of medical approaches by deepening society's understanding of HIV and supporting people living with HIV to tell the stories of their communities [4, 5]. The initiative, across two editions of the programme, included a total of 24 artists from diverse age groups and regions around the world. Among them, two youth artists—Kairon Liu from the Asia‐Pacific region and Ismail Senyonga from the African region—not only use their art to raise public awareness, but also to demonstrate how artistic expression can serve as a form of self‐reflection. Through their work, they document the path of living with HIV and translate these experiences into powerful narratives of self‐care and resilience.

“Untransmittable,” exhibited in 2023, featured Kairon's repurposing of expired antiretroviral medications donated by the Taiwanese HIV community, revealing the complex social meanings embedded within biomedical materials while delivering updated HIV knowledge. The photograph visually references the medical advancements that have rendered HIV untransmittable when managed with consistent ART. However, it also quietly signals an uncomfortable truth: despite the availability of antiretroviral medicines, economic and psychological burdens continue to weigh heavily on people. Scientific advancements can still be inaccessible because of personal and structural barriers, leaving a good quality of life out of reach for many. This remains undeniably evident even now, as numerous HIV treatment and prevention options exist, yet equitable access for those in need is still lacking.

“Alone and Frightened,” “Circle of Love,” and “Acceptance,” presented in 2024, are a series of paintings by Ismail Senyonga that emphasize the urgent need for better paediatric medicines and highlight the complex transition from childhood to adulthood. For young people living with HIV, especially those who acquired it perinatally, this period is often marked by fear, anxiety, depression, and even suicidal ideation. The downcast and exhausted body language and expressions of the figures in these paintings powerfully capture the candid, emotional realities faced by young people living with HIV as they navigate their personal challenges and overcome the anxieties of growing up in their societies.

These profoundly emotional pieces are not meant to portray young people living with HIV as merely vulnerable; rather, they strike at the heart of a deeper issue—the persistent inequalities that youth face within our society. Whether it is unequal access to healthcare resources or the lack of social acceptance and inclusivity, these works serve as a powerful call for urgent societal change. The driving force behind this call for transformation is none other than the hopeful and courageous generation of young people themselves.

On this International Youth Day, we encourage all stakeholders to recommit resources towards a youth‐centred HIV response. This includes meaningfully involving young people in decision‐making spaces related to the equitable distribution of medical resources, healthcare system strengthening, economic empowerment, mental health support and the decriminalization of key populations. Young people bring unique insights into effective service delivery models, community engagement strategies and stigma reduction approaches. Their perspectives must be integral to programme design, resource allocation and policy development at local, national and international levels.

Youth representation within major international organizations—like IAS Youth Hub and Global Fund Youth Council—should continue to be prioritized to strengthen their capacity to actively engage and lead in these spaces. We highlighted two youth artists today to remind us of the vulnerability of young people to HIV and the transcendent ability of ART to remind us of our shared values. We will continue to stimulate discussion and new thinking that inspire those around us to learn about HIV and shift the narrative—from risk to intimacy, and from pathology to humanity. By embracing these comprehensive strategies, the HIV movement can achieve lasting progress—empowering us not only to meet the UNAIDS 2030 targets but also to sustain hope and confidence for a future well beyond those milestones.

## COMPETING INTERESTS

The authors declare no competing interests.

## AUTHORS’ CONTRIBUTIONS

FC, KL and IS contributed to the writing and review of the article.

## AUTHOR INFORMATION

The artworks referenced in the article are accessible via the following links. Untransmittable: www.kaironliu.com/untransmittable.

## Data Availability

Data sharing is not applicable to this article as no new data were created or analysed in this study.
